# A cosmetic ‘anti-ageing’ product improves photoaged skin: a double-blind, randomized controlled trial

**DOI:** 10.1111/j.1365-2133.2009.09216.x

**Published:** 2009-08

**Authors:** REB Watson, S Ogden, LF Cotterell, JJ Bowden, JY Bastrilles, SP Long, CEM Griffiths

**Affiliations:** Dermatological Sciences Research Group, School of Translational Medicine, Faculty of Medical and Human Sciences, The University of ManchesterOxford Road, Manchester M13 9PT, U.K.; * Alliance Boots LtdNottingham NG2 3AA, U.K.

**Keywords:** fibrillin-1, patch-test assay, randomized controlled trial, wrinkles

## Abstract

**Background:**

Very few over-the-counter cosmetic ‘anti-ageing’ products have been subjected to a rigorous double-blind, vehicle-controlled trial of efficacy. Previously we have shown that application of a cosmetic ‘anti-ageing’ product to photoaged skin under occlusion for 12 days can stimulate the deposition of fibrillin-1. This observation infers potential to repair and perhaps clinically improve photoaged skin.

**Objective:**

We examined another similar over-the-counter cosmetic ‘anti-ageing’ product using both the patch test assay and a 6-month double-blind, randomized controlled trial (RCT), with a further 6-month open phase to assess clinical efficacy in photoaged skin.

**Methods:**

For the patch test, a commercially available test product and its vehicle were applied occluded for 12 days to photoaged forearm skin (*n*=10) prior to biopsy and immunohistochemical assessment of fibrillin-1; all-*trans* retinoic acid (RA) was used as a positive control. Sixty photoaged subjects were recruited to the RCT (test product, *n* = 30 vs. vehicle, *n* = 30; once daily for 6 months, face and hands) with clinical assessments performed at recruitment and following 1, 3 and 6 months of use. Twenty-eight volunteers had skin biopsies (dorsal wrist) at baseline and at 6 months treatment for immunohistochemical assessment of fibrillin-1 (test product, *n*=15; vehicle, *n*=13). All volunteers received the test product for a further 6 months. Final clinical assessments were performed at the end of this open period.

**Results:**

In the 12-day patch test assay, we observed significant immunohistological deposition of fibrillin-1 in skin treated with the test product and RA compared with the untreated baseline (*P*=0·005 and 0·015, respectively). In the clinical RCT, at 6 months, the test product produced statistically significant improvement in facial wrinkles as compared to baseline assessment (*P* = 0·013), whereas vehicle-treated skin was not significantly improved (*P* = 0·11). After 12 months, there was a significant benefit of the test product over that projected for the vehicle (70% vs. 33% of subjects improving; combined Wilcoxon rank tests, *P*=0·026). There was significant deposition of fibrillin-1 in skin treated for 6 months with the test product [(mean ± SE) vehicle 1·84 ± 0·23; test product 2·57 ± 0·19; ancova*P*=0·019).

**Conclusions:**

In a double-blind RCT, an over-the-counter cosmetic ‘anti-ageing’ product resulted in significant clinical improvement in facial wrinkles, which was associated with fibrillin-1 deposition in treated skin. This study demonstrates that a cosmetic product can produce significant improvement in the appearance of wrinkles and further supports the use of fibrillin-1 as a robust biomarker for the repair of photoaged dermis.

All tissues, regardless of body site, are subject to intrinsic ageing, the result of the passage of time. Few clinically apparent changes occur in intrinsically aged skin until the individual is over 70 years of age at which point fine wrinkles become apparent.^1^ Skin, more than any other organ, is also subject to environmental influences which can lead to extrinsic ageing. One such environmental factor is chronic exposure to sunlight which results in phenotypic changes termed photoageing—inevitably a combination of intrinsic ageing and photodamage. By comparison with intrinsic ageing, photoaged skin is rough, dyspigmented and exhibits both fine and deep wrinkles.^2^,^3^ Histological examination of intrinsically aged skin reveals atrophy of the dermal extracellular matrix (ECM), with reduced levels of collagen and elastin.^4^ Photoaged skin has a different ECM morphology with solar elastosis—the deposition of dystrophic elastic fibres in the dermis—being a prominent histological feature.^5^ Photoaged dermis contains significantly reduced levels of collagen types I and III,^6^ fewer anchoring fibrils at the dermal–epidermal junction (DEJ; collagen VII)^7^ and loss of the fibrillin-rich microfibrillar architecture in the papillary dermis.^8^ These remodelled ageing phenotypes are thought in part to be due to increased cutaneous expression of matrix metalloproteinases (MMPs).^9^–^11^

Topical retinoids are used as the clinical, evidence-based ‘gold standard’ for the treatment of photoaged skin.^12^ Numerous studies have shown the reparative effects of topical application of all-*trans* retinoic acid (RA), which includes the partial restoration of collagens I, III^13^ and VII^14^ and restoration of the fibrillin-rich microfibrillar network.^15^ These ECM changes, together with reduced MMP expression may in part explain the clinical improvement of photoaged skin produced by topical retinoids.^16^–^18^ We showed previously, in a 12-day occluded patch test assay, that a specific cosmetic ‘anti-ageing’ product also has the ability to stimulate the accumulation of fibrillin-1.^19^

Although prescription retinoids can affect these significant clinical and histological changes in photoaged skin there is scant evidence that any of the plethora of cosmetic ‘anti-ageing’ products can produce similar effects. We firstly examined whether another, similar cosmetic ‘anti-ageing’ product can induce accumulation of fibrillin-1 in photoaged skin using the patch test protocol. We then investigated the same product in a rigorous double-blind, randomized controlled trial (RCT) to ascertain whether or not its use results in a clinically detectable benefit.

## Methods

### Test products

A commercially available product provided by Alliance Boots Ltd (No7 Protect & Perfect Intense Beauty Serum™; Alliance Boots Ltd, Nottingham, UK) was investigated in these studies, together with a vehicle formulation. The product is a water in silicone emulsion with glycerine and other emollients and a complex of ‘anti-ageing’ ingredients comprising natural extracts and peptides: sodium ascorbyl phosphate, *Panax ginseng*, *Morus alba*, *Lupinus alba*, tocopherol, palmitoyl oligopeptide, palmitoyl tetrapeptide-7, *Medicago sativa* and retinyl palmitate. The vehicle was of identical composition, but without the complex of ‘anti-ageing’ ingredients.

### *In vivo* patch test study

Ten healthy but photoaged volunteers were recruited (four men, six women; age range 61–76 years) and subjected to an extended 12-day patch test assay.^19^ Test substances (vehicle and test product 20 μL) were applied separately to the extensor photoaged aspect of the forearm under standard 6-mm diameter Finn chambers (Scanpore, Tuulsula, Finland). In addition, an area was left untreated but was occluded to provide a baseline control sample. Test products were applied to clean skin on days 1, 4 and 8 of the assay. RA (0·025%; Retin-A® cream; Janssen-Cilag Ltd, Beerse, Belgium; 20 μL) was applied to an untreated site on day 8 and left *in situ* for 4 days to avoid potential complications of irritancy caused by extended occlusion. On day 12, the Finn chambers were removed and 3-mm punch biopsies were obtained under 1% lignocaine local anaesthesia from each test site. Biopsied tissue was embedded in optimal cutting temperature compound (Tissue-Tek®; Miles Laboratories, Elkhart, IN, U.S.A.), snap frozen in liquid nitrogen and stored at −70 °C prior to immunohistochemical analyses. The North Manchester Local Research Ethics Committee approved the study and all subjects gave written, informed consent.

### Slide preparation

Frozen sections were prepared at a thickness of 10 μm (OTF cryostat; Bright Instruments Ltd, Cambridge, U.K.) and mounted onto gelatin-coated slides prior to histological analysis.

### Immunohistochemistry

Immunohistochemistry was performed as previously described^19^ to identify a panel of ECM molecules or remodelling enzymes in frozen sections from the 12-day patch test assay and from the RCT. Primary antibodies were applied overnight at 4 °C. These were: mouse antihuman fibrillin-1 (clone 11C1.3; Neomarkers, Union City, CA, U.S.A.) diluted 1 : 100; rat antihuman procollagen-1 (pCI) (clone M-58; Chemicon International Inc., Temecula, CA, U.S.A.) diluted 1 : 1000; or mouse antihuman MMP-1 (Oncogene Research Products, Boston, MA, U.S.A.) diluted 1 : 100. Negative controls were by incubation of isotype sera at the appropriate concentration or omission of the primary antibody. Sections were washed in TBS prior to incubation with the appropriate biotinylated secondary antibody for 30 min. Antibody staining was visualized using a well-characterized immunoperoxidase reaction (VectaStain®*Elite* ABC system; Vector Laboratories, Burlingame, CA, U.S.A.) utilizing Vector SG® as chromogen. Following light counterstaining with nuclear fast red, sections were serially dehydrated and permanently mounted. Stained sections were randomized, blinded and examined on a Nikon OPTIPHOT microscope (Tokyo, Japan). The degree of immunostaining for fibrillin-1 and pCI was assessed as previously described.^8^,^15^,^19^ In brief, a five-point semiquantitative scale was used where 0 = no staining and 4 = maximal staining within the experiment. The numbers of epidermal keratinocytes positive for MMP-1 were quantified per high-power field (hpf; × 400). Four sections (including control) were examined per subject, per site, per treatment and the average score calculated.

### Randomized controlled trial

Sixty healthy but photoaged volunteers were recruited to this study (11 men, 49 women; age range 45–80 years). All test products were supplied in identically packaged, coded containers so that the investigators and subjects were unaware as to the treatment. Subjects were randomly allocated to self-treatment with either the vehicle formulation or the test product as described by a randomization programme (StatsDirect Ltd, Altrincham, U.K.) and instructed on the use of their allotted cream—daily evening application to the entire face and dorsa of the hands, including the wrists and extensor forearm, for 6 months. Clinical assessments of the skin of the face and dorsal hands were performed for all participants at baseline and following 1, 3 and 6 months of product use. The following four parameters were assessed at each visit: fine lines and wrinkles, dyspigmentation, overall clinical grade of photoageing and tactile roughness. The degree of fine lines and wrinkles, dyspigmentation and the overall level of photoageing were scored according to the well-characterized Griffiths photonumeric scale for photoaged skin.^20^ The scale ranges from 0 to 8, where 0 represents no evidence of photoageing and 8 represents the most severe photoageing. Pigment was assessed on a similar 0–8 scale, where 0 denotes a uniform coloration of the skin with absence of photoageing-related colour change and 8 represents severe dyspigmentation. Likewise, tactile roughness was scored on the treated areas from 0 to 8, where 0 represents totally smooth skin with no rough patches and 8 represents very roughened skin.

In addition, 28 subjects provided 3-mm skin biopsies from the dorsal wrist at the beginning and end of the 6-month study period (vehicle formulation, *n*=13; test product, *n*=15). These biopsies were evaluated for the expression of fibrillin-1 in the papillary dermis, as previously described.^19^ All the subjects were monitored for the occurrence of serious adverse events up to, and including, 28 days after their involvement with this study. The Salford and Trafford Local Research Ethics Committee approved the study and all the subjects gave written, informed consent.

### Statistical analyses

#### *In vivo* patch test study

Differences in the amount of fibrillin-1 immunostaining produced by the vehicle and test product were assessed for significance using the repeated measures analysis of variance (anova). Results were considered significant if *P*<0·05 (95% confidence level) and were calculated using SPSS+ v 11.5 software (SPSS Inc., Chicago, IL, U.S.A.).

##### Clinical assessment

Analysis of covariance (ancova), using the baseline as covariate, was used to assess the 6-month data. Linear regression analysis was used to extrapolate the vehicle response to 12 months, thus allowing direct comparison with the test product, validity confirmed by the Monte Carlo simulation.^21^ As all volunteers used the test product in the final 6 months, the 12-month clinical assessment data were analysed using a combination of Wilcoxon’s matched pairs signed rank and rank sum tests, to give an overall *P*-value.

##### In-use biopsy samples

ancova was performed, using the baseline as covariate to assess changes in the deposition of fibrillin-1 in the papillary dermis, with significance taken at the 95% confidence level (SAS 9.1; SAS Institute Inc., Cary, NC, U.S.A.).

## Results

### *In vivo* patch test study

**Fig 1:**
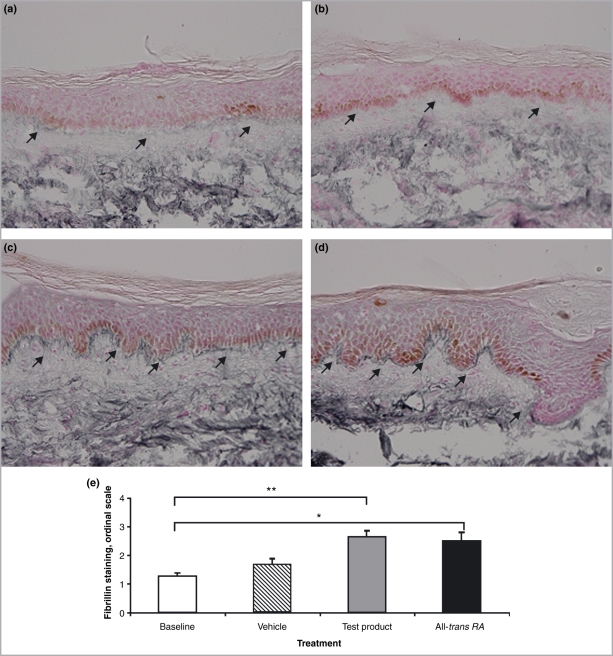
Fibrillin-rich microfibrils are deposited in test product- and all-*trans* retinoic acid (RA)-treated human skin in a short-term patch test assay. Representative photomicrographs showing fibrillin-rich microfibrils (arrows) in photoaged extensor forearm following the extended 12-day patch test protocol: (a) baseline; (b) vehicle; (c) test product. (d) The positive control (0·025% RA) was applied for 4 days to avoid harmful side-effects. Original magnification × 400. (e) Quantification of fibrillin-rich microfibrils following the occluded patch test assay with vehicle, test product or the positive control (RA), compared with the baseline. We identified significantly more fibrillin-rich microfibrils with both the test product (**P=0·005) and RA (*P=0·015).

RA (the clinical ‘gold standard’) produced a significant deposition of fibrillin-1 in the papillary dermis compared with that observed at baseline (*P*=0·015). Application of the vehicle, following the 12-day patch test assay, produced little effect on fibrillin-1 deposition (*P*>0·05). However, application of the test product resulted in a significant deposition of fibrillin-1, the accumulation being at a similar level to that observed using RA (mean ± SE) (baseline 1·27 ± 0·11; vehicle formulation 1·70 ± 0·17; test product 2·64 ± 0·22, *P*=0·005; RA 2·51 ± 0·28, *P*=0·015; Fig. 1e). As in previous studies,^15^ treatment with RA had little effect on deposition of pCI or on the expression of MMP-1 in the epidermis (Table 1).

**Table 1:** Expression of extracellular matrix (ECM) molecules in photoaged skin following 12-day occlusion with a cosmetic ‘anti-ageing’ product

ECM component	Baseline	Vehicle	Test product	0·05% RA
Fibrillin-1	1·27 ± 0·11	1·70 ± 0·17	2·64 ± 0·22**	2·51 ± 0·28*
pCI	2·65 ± 0·15	2·64 ± 0·15	2·70 ± 0·13	2·63 ± 0·21
MMP-1 (cells/hpf)	115·8 ± 7·9	140·3 ± 13·6	127·2 ± 14·1	143·7 ± 12·5

RA, all-*trans* retinoic acid; pCI, procollagen-1; MMP, matrix metalloproteinase; hpf, high-power field.

* *P*=0·015;

** *P*=0·005.

### Randomized controlled trial

#### Clinical assessment

**Fig 2:**
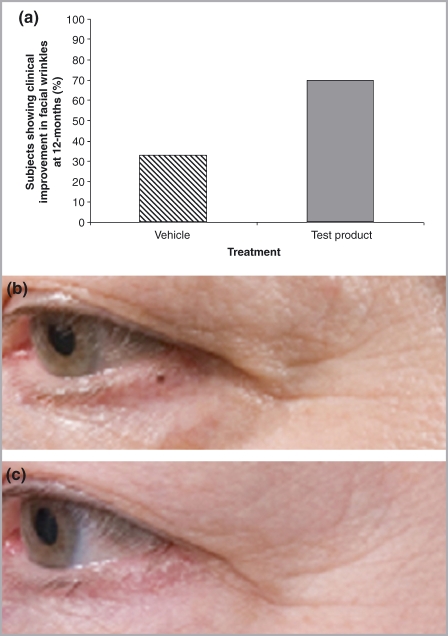
Application of the test product results in clinical improvement in facial wrinkles. (a) Clinical assessment identified a significant improvement (*P*=0·026) in wrinkle grade following treatment with the test product compared with the vehicle at 12 months, as ascertained by using a Monte Carlo simulation. Standardized photographs of the face of a 56-year-old woman at (b) baseline and (c) after 6 months’ application of the test product show an improvement in periorbital fine lines and wrinkles.

At 6 months, the test product produced statistically significant improvement in facial wrinkles as compared to baseline assessment (*P* = 0·013), whereas vehicle-treated skin was not significantly improved (*P* = 0·11). 43% of the subjects who had received the test product showed an improvement in facial wrinkles compared with the baseline assessment, whereas only 22% of the subjects receiving the vehicle showed improvement compared with baseline (Fig. 2). Use of the test product produced a clinically significant improvement in facial wrinkles after 12 months of use, with a statistically significant between-groups benefit of test product vs. the vehicle (test product, 70% of subjects improving compared with vehicle, 33% improving; combined Wilcoxon rank tests, *P*=0·026) (Fig. 2). No benefits of the test product were seen for improvement in mottled dyspigmentation. Use of either formulation produced an improvement in skin texture over that recorded at baseline (vehicle, *P*=0·001; test product, *P*=0·001), but the test formulation did not perform significantly better than the vehicle (data not shown; *P*=0·72).

#### In-use biopsy samples

**Fig 3:**
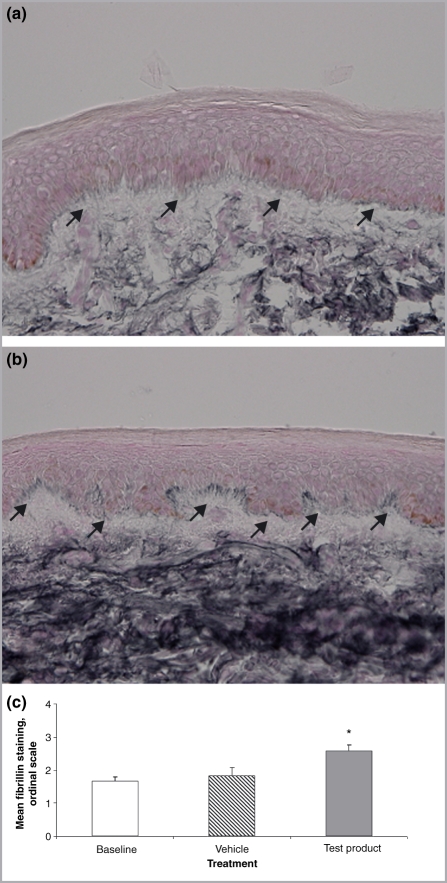
Fibrillin-rich microfibrils were assessed by immunohistochemistry at recruitment (baseline, a) and after treatment with either vehicle (data not shown) or test product (b). (c) Significant deposition of fibrillin-rich microfibrils was observed in the test product-treated skin. *P=0·019.

The test product produced a significant accumulation of fibrillin-1 in the papillary dermis of photoaged skin at 6 months compared with the vehicle (mean ± SE) (vehicle formulation 1·84 ± 0·23; test product 2·57 ± 0·19; ancova, *P*=0·019; Fig. 3).

## Discussion

We show here, for the first time, that a commercially available over-the-counter ‘anti-ageing’ product improves the appearance of facial wrinkles when used in the long term. This improvement is associated with restoration of fibrillin-1, the major component of fibrillin-rich microfibrils, in product-treated skin.

In these studies, we performed a double-blind RCT to assess whether or not an over-the-counter cosmetic ‘anti-ageing’ product can produce clinically significant improvement in the appearance of photoaged facial skin. The trial was executed to the highest standards, with study creams coded and randomized at source, and with the volunteers, investigators and independent statistician ‘blind’ to the coding until after study completion and initial data analysis. We did not observe any benefit of the vehicle or test product on the appearance of mottled dyspigmentation or actinic lentigines associated with photoageing. Expert clinical assessments (S.O., J.Y.B.) showed that subjects treated with the vehicle formulation had improved skin texture compared with that observed at their recruitment, but did not exhibit any change in the appearance of their facial wrinkles. Those treated with the test product showed improvements in both skin texture (compared with their baseline clinical assessment) and, more importantly, in the appearance of facial wrinkles. This improvement in the appearance of facial wrinkles became significant only after 12 months of daily product use comparing between groups. It is interesting to note, however, that when compared with the baseline, the test product did lead to a noticeable clinical improvement in facial wrinkles (*P* = 0·013) in 43% of treated individuals after 6 months, compared with only 22% of those treated with the vehicle where there was no significant improvement in appearance (*P* = 0·11). In a comparison between groups, this improvement was not statistically significant but does indicate that larger clinical trials of cosmetic products might be expected to show useful clinical improvement after 6 months’ use. The data from this study are indicative that cosmetic ‘anti-ageing’ products can result in noticeable clinical improvement in facial wrinkles. To our knowledge, this is the first time such benefits have been reported for a commercially available cosmetic ‘anti-ageing’ product and paves the way for larger studies with more statistical power.

In addition, we examined the distribution of a key biomarker of photoageing—loss of fibrillin-1 in the papillary dermis—in skin samples obtained during the RCT. Fibrillin-1 is the major glycoprotein component of fibrillin-rich microfibrils (oxytalan fibres). Skin treated with the test product contained significantly more fibrillin-rich microfibrils in the papillary dermis than either the baseline samples or those treated with the vehicle (*P*=0·019). Our previous research, which demonstrated that an over-the-counter cosmetic ‘anti-ageing’ product can bring about increased fibrillin-1 deposition in the papillary dermis, used a short-term, exaggerated-use patch test assay.^19^ In the current study, we sought to determine whether results obtained using such an experimental system were predictive of clinical improvement following long-term use of such a product.

Consumers purchasing cosmetic skin care products—particularly those purporting ‘anti-ageing’ properties—are presented with a broad choice of available products and only limited data regarding their efficacy. The situation is further complicated for consumers by the use of trademarked proprietary names for ingredients and the relative lack of published long-term studies to demonstrate product performance. We previously sought to investigate if a cosmetic ‘anti-ageing’ product can have a measurable effect on fibrillin-1 and established that histological improvement could be achieved in a short-term exaggerated-use assay.^19^ The results from that study were indicative of some degree of structural change in the skin following use of a cosmetic but provided no evidence for actual clinical improvement. To address this question, the current study compared the use of a commercially available cosmetic ‘anti-ageing’ skincare serum to its vehicle and has shown that such a product is capable of bringing about a clinical improvement in the appearance of photoaged skin when used for 12 months.

The difference in efficacy between the vehicle and the test product demonstrates that a correctly formulated skincare product can deliver clinically relevant skin improvement, above that delivered by the vehicle base. The studied product contains the retinol ester, retinyl palmitate, together with natural plant extracts, peptides and lipopeptides and antioxidants. Other authors have shown evidence for the role of many of these cosmetic ingredients in protecting against mechanisms that lead to dermal degradation, such as increased MMP activity^22^ and stimulating repair of dermal components.^23^–^25^ It was our belief that a combination of ingredients with activities known to address the multiple changes which occur in photoaged skin (degradation of collagen and elastin, the appearance of surface wrinkling and textural changes) may be beneficial in a cosmetic product when used long term. Specifically, the retinyl palmitate, palmitoyl peptides and *Medicago sativa* extracts have been shown *in vitro* to lead to deposition of collagen 1 in model skin systems and the extract of white lupin has been shown to inhibit MMP-1 (S.P. Long, unpublished data). In our previous study using the 12-day assay, a similar product induced deposition of pCI as well as fibrillin, together with inhibition of MMPs.^19^ These data suggest that the long-term benefits described here may be due to a combination of actions. The product does not contain sunscreens, so the visible skin improvements cannot be attributed to photoprotection of the skin. Further work is underway to elucidate the relative contributions of the individual ingredients.

The finding that a clinically relevant improvement in the appearance of photoaged skin was demonstrated with long-term use of a commercially available cosmetic ‘anti-ageing’ product may cause some to question whether such effects are within the definition of a cosmetic. Improvement in the appearance of wrinkles is considered to be a cosmetic action, as the effect is localized to the skin and is not concerned with treatment or correction of a disease condition. Several other authors report that application of cosmetic products leads to changes in skin physiology, including changes in barrier function,^26^,^27^ stratum corneum thickness^28^ and lipidogenesis^29^ and that cumulative effects of cosmetic products are possible, a principle demonstrated for skin moisturization^30^ and recognized by regulatory authorities.^31^ When compared with the long-term clinical effects of topical RA, it can be seen that the degree of improvement offered by a cosmetic product is still markedly less than that which is achievable with a prescribed medicine.^32^–^38^

In conclusion, these studies provide evidence that use of an over-the-counter cosmetic ‘anti-ageing’ product is able to induce clinically identifiable improvement in the appearance of facial wrinkles following long-term use. This improvement is associated with deposition of fibrillin-rich microfibrils in the papillary dermis of treated skin. The study further supports the use of fibrillin-1 in a short-term assay as a biomarker for assessing efficacy of potential photoageing repair products.

## References

[1] Lavker RM, Zheng PS, Dong G (1987). Aged skin: a study by light, transmission electron, and scanning electron microscopy. J Invest Dermatol.

[2] Smith JG, Davidson EA, Sams WM (1962). Alterations in human dermal connective tissue with age and chronic sun damage. J Invest Dermatol.

[3] Warren R, Gartstein V, Kligman AM (1991). Age, sunlight, and facial skin: a histologic and quantitative study. J Am Acad Dermatol.

[4] Braverman IM, Fonferko E (1982). Studies in cutaneous aging: I. The elastic fiber network. J Invest Dermatol.

[5] Chen VL, Fleischmajer R, Schwartz E (1986). Immunochemistry of elastotic material in sun-damaged skin. J Invest Dermatol.

[6] Talwar HS, Griffiths CEM, Fisher GJ (1995). Reduced type I and type III procollagens in photodamaged adult human skin. J Invest Dermatol.

[7] Craven NM, Watson REB, Jones CJ (1997). Clinical features of photodamaged human skin are associated with a reduction in collagen VII. Br J Dermatol.

[8] Watson REB, Griffiths CEM, Craven NM (1999). Fibrillin-rich microfibrils are reduced in photoaged skin. Distribution at the dermal–epidermal junction. J Invest Dermatol.

[9] Varani J, Warner RL, Gharaee-Kermani M (2000). Vitamin A antagonizes decreased cell growth and elevated collagen-degrading matrix metalloproteinases and stimulates collagen accumulation in naturally aged human skin. J Invest Dermatol.

[10] Chung JH, Seo JY, Choi HR (2001). Modulation of skin collagen metabolism in aged and photoaged human skin *in vivo*. J Invest Dermatol.

[11] Brennan M, Bhatti H, Nerusu KC (2003). Matrix metalloproteinase-1 is the major collagenolytic enzyme responsible for collagen damage in UV-irradiated human skin. Photochem Photobiol.

[12] Samuel M, Brooke RCC, Hollis S, Griffiths CEM (2005). Interventions for photoaged skin. Cochrane Database Syst Rev.

[13] Griffiths CEM, Russman AN, Majmudar G (1993). Restoration of collagen formation in photodamaged human skin by tretinoin (retinoic acid). N Engl J Med.

[14] Woodley DT, Zelickson AS, Briggaman RA (1990). Treatment of photoaged skin with topical tretinoin increases epidermal–dermal anchoring fibrils. A preliminary report. JAMA.

[15] Watson REB, Craven NM, Kang S (2001). A short-term screening protocol, using fibrillin-1 as a reporter molecule, for photoaging repair agents. J Invest Dermatol.

[16] Fisher GJ, Talwar HS, Lin J (1999). Molecular mechanisms of photoaging in human skin *in vivo* and their prevention by all-trans retinoic acid. Photochem Photobiol.

[17] Watson REB, Ratnayaka AJ, Brooke RCC (2004). Retinoic acid receptor alpha expression and cutaneous ageing. Mech Ageing Dev.

[18] Lateef H, Stevens MJ, Varani J (2004). All-*trans* retinoic acid suppresses matrix metalloproteinase activity and increases collagen synthesis in diabetic human skin in organ culture. Am J Pathol.

[19] Watson REB, Long SP, Bowden JJ (2008). Repair of photoaged dermal matrix by topical application of a cosmetic ‘antiageing’ product. Br J Dermatol.

[20] Griffiths CEM, Wang TS, Hamilton TA (1992). A photonumeric scale for the assessment of cutaneous photodamage. Arch Dermatol.

[21] Chakrapani C (1994). Meta Analysis 1: How to Combine Research Studies.

[22] Schroeder P, Lademann J, Darvin ME (2008). Infrared radiation-induced matrix metalloproteinase in human skin: implications for protection. J Invest Dermatol.

[23] Humbert PG, Haftek M, Creidi P (2003). Topical ascorbic acid on photoaged skin. Clinical, topographical and ultrastructural evaluation: double-blind study vs. placebo. Exp Dermatol.

[24] Talbourdet S, Sadick NS, Lazou K (2007). Modulation of gene expression as a new skin anti-aging strategy. J Drugs Dermatol.

[25] Haftek M, Mac-Mary S, Le Bitoux MA (2008). Clinical, biometric and structural evaluation of the long-term effects of a topical treatment with ascorbic acid and madecassoside in photoaged human skin. Exp Dermatol.

[26] Chang MJ, Huang HC, Chang HC (2008). Cosmetic formulations containing *Lithospermum erythrorhizon* root extract show moisturizing effects on human skin. Arch Dermatol Res.

[27] Puglia C, Bonina F (2008). *In vivo* spectrophotometric evaluation of skin barrier recovery after topical application of soybean phytosterols. J Cosmet Sci.

[28] Crowther JM, Sieg A, Blenkiron P (2008). Measuring the effects of topical moisturizers on changes in stratum corneum thickness, water gradients and hydration *in vivo*. Br J Dermatol.

[29] Bazin R, Fanchon C (2006). Equivalence of face and volar forearm for the testing of moisturizing and firming effect of cosmetics in hydration and biomechanical studies. Int J Cosmet Sci.

[30] Kligman A (1978). Regression method for assessing the efficacy of moisturisers. Cosmet Toiletries.

[31] ASA Compliance Report (2007). Cosmetics Advertising Survey.

[32] Kligman AM, Grove GL, Hirose R (1986). Topical tretinoin for photoaged skin. J Am Acad Dermatol.

[33] Weiss JS, Ellis CN, Headington JT (1988). Topical tretinoin improves photoaged skin. A double-blind vehicle-controlled study. JAMA.

[34] Ellis CN, Weiss JS, Hamilton TA (1990). Sustained improvement with prolonged topical tretinoin (retinoic acid) for photoaged skin. J Am Acad Dermatol.

[35] Rafal ES, Griffiths CEM, Ditre CM (1992). Topical tretinoin (retinoic acid) treatment for liver spots associated with photodamage. N Engl J Med.

[36] Griffiths CEM, Voorhees JJ (1993). Topical retinoic acid for photoaging: clinical response and underlying mechanisms. Skin Pharmacol.

[37] Griffiths CEM, Goldfarb MT, Finkel LJ (1994). Topical tretinoin (retinoic acid) treatment of hyperpigmented lesions associated with photoaging in Chinese and Japanese patients: a vehicle-controlled trial. J Am Acad Dermatol.

[38] Griffiths CEM (2001). The role of retinoids in the prevention and repair of aged and photoaged skin. Clin Exp Dermatol.

